# Phosphoprotein expression profiles in rat kidney injury: Source for potential mechanistic biomarkers

**DOI:** 10.1111/jcmm.14103

**Published:** 2019-01-12

**Authors:** Vitalina Gryshkova, Mabel Cotter, Portia McGhan, Jana Obajdin, Renaud Fleurance, Andre Nogueira da Costa

**Affiliations:** ^1^ Investigative Toxicology, Development Science UCB Biopharma SPRL Braine L'Alleud Belgium; ^2^ Safety & Environmental Assurance Centre, Unilever U.K. London UK; ^3^ MRC Institute of Genetics & Molecular Medicine The University of Edinburgh, Western General Hospital Edinburgh UK; ^4^ Centre for Stem Cells & Regenerative Medicine King's College London London UK; ^5^ Experimental Medicine and Diagnostics, Translational Medicine UCB Biopharma SPRL Braine L'Alleud Belgium

**Keywords:** biomarkers, kidney injury, nephrotoxicity, phosphorylation

## 1 INTRODUCTION

Acute kidney injury (AKI) is defined by the Acute Kidney Injury Network as “functional and structural disorder or signs of renal damage including any defect from blood or urine test, or tissue imaging that is less than 3 months.” AKI is the most common cause of renal dysfunction.^1^ Understanding of molecular determinants of AKI induction and development could help to diagnose and monitor kidney injury in the clinic as well as to select safe drug candidates in preclinical drug development.

Protein phosphorylation plays a significant role in a wide range of cellular processes and can directly indicate an active/inactive state of cellular enzymes and the signalling pathways in injury initiation and progression. Alteration of phosphorylation of some proteins was already described for kidney injury: MAPK activation in cisplatin‐induced nephrotoxicity,^2^ increased phosphorylation of moesin and HSP90ɑ in tubular cells in response to TGF‐β^3^ and increased phosphorylation of lamin A and phospholamban in a salt‐load rat model of kidney damage.^4^

Our study aimed at identifying changes in phosphoprotein expression and creating comprehensive phosphoprotein profiles of nephron‐specific injuries in a time‐dependent manner. To meet this objective, we utilized rat models of drug‐induced kidney injury (DIKI), which were induced with three nephrotoxicants over a 28‐day period: cisplatin (proximal tubules); puromycin (glomerulus) and *N*‐phenylanthranylic acid (NPAA, collecting ducts). The phosphoproteins were studied by antibody microarrays in kidney tissue on days 7, day 14 and day 28. We established changes in phosphoprotein expression in response to kidney injury and associated these changes with biological processes. Up‐regulation of MEK2 (pThyr394) on day 14 in cisplatin and puromycin‐induced injuries was confirmed by immunohistochemistry (IHC). Identified phosphoprotein signatures provide a link to molecular mechanisms of kidney injury/recovery as well as a source for potential mechanistic biomarkers.

## 2 MATERIALS AND METHODS

### 2.1 In vivo study design and kidney collection

The study design is described in detail by Obajdin et al.^5^ Briefly, Hannover Wistar rats were treated with: single oral administration of cisplatin, daily oral dosing with puromycin, NPAA or vehicle controls (VC1 [0.9% NaCl] or VC2 [1.25% carboxymethyl cellulose]) over 28 days. Six animals per treatment group were killed on days 7, 14 and 28. Collected kidneys were used for protein profiling. All procedures were approved by the ethical committee for animal experimentation at UCB Biopharma SPRL and were in accordance with the European Directive 2010/63/EU.

### 2.2 PhosphoExplorer array

PhosphoExplorer Array (Full Moon Biosystems, SEV03‐PEX100) was used to perform phosphoprotein profiling in kidney tissue. Three kidney tissue samples per treatment/per time‐point were selected in a blinded manner. Protein extraction, labelling, coupling and detection were performed according to manufacturer's recommendations. The slides were scanned on InnoScan 710 (Innopsys, France) and processed with Mapix software (Innopsys, France).

### 2.3 Data analysis

The signal intensities of phosphorylated proteins were normalized to non‐phosphorylated proteins and the rations were compared between treatments and vehicle controls to calculate fold changes (FC) at matched time‐points using *t* test. The fold change of 1.5 and *P *≤ 0.05 was applied to select significantly dysregulated phosphoproteins. To analyse the implication of selected candidates in different biological processes, PhosphoSitePlus was utilized.

### 2.4 Immunohistochemical (IHC) analysis

Four micrometre formalin‐fixed paraffin embedded kidney sections were stained with MEK2 (pThr394) antibody (Abcam, ab30622). Immunostaining was performed by using an Omni Map DAB kit (Roche, 760‐4310). Slides were scanned using a NanoZoomer XR scanner (Hamamatsu, Japan) at a 20× magnification for whole slide imaging and visualized using NDP.view 2 software (Hamamatsu, Japan).

## 3 RESULTS

### 3.1 Phosphoprotein profiling in rat models of DIKI

Administration of cisplatin, puromycin or NPAA‐induced kidney injuries specific to proximal tubule, glomerulus (with secondary tubular dilation) or collecting duct, respectively, which was confirmed by histopathological assessment and urinary biomarkers previously.^5^ Using phosphoprotein/protein microarrays, we evaluated protein phosphorylation in the kidney and selected significantly dysregulated candidates (0.7 ≤ FC ≥ 1.5, *P* ≤ 0.05) for each treatment at three time‐points (days 7, 14 and 28) (Table S1–S3). Table 1 shows treatment‐specific phosphoprotein signatures of rat kidney injury in a time‐dependent manner. Among selected phosphoproteins, MEK2 (pThr394) had the highest FC after cisplatin and puromycin administration on day 14 compared to vehicle controls (2.2x and 2.5x respectively) (Table S2).

**Table 1:** Changes in phosphoprotein expression in kidney tissue in response to nephrotoxicants during 28‐day study

D7		D14		D28	
Phosphoprotein	FC	Phosphoprotein	FC	Phosphoprotein	FC
*Cisplatin*					
p53 (p‐Ser378)	2	MEK2 (p‐Thr394)	2.2	c‐Jun (p‐Thr93)	1.7
NFkB‐p65 (p‐Ser468)	1.8	Synuclein α(p‐Tyr125)	1.8	P70S6K (p‐Ser371)	0.5
GSK3β (p‐Ser9)	1.8	AKT1 (p‐Thr308)	1.6	PAK1/2/3 (p‐Thr423/402/421)	0.5
IRS‐1 (p‐Ser323)	1.8	Src (p‐Tyr529)	1.6	IKK‐α (p‐Thr23)	0.5
SMC1 (p‐Ser957)	1.7	EGFR (p‐Tyr869)	0.5	GATA1 (p‐Ser310)	0.6
LCK (p‐Tyr504)	1.7	Claudin 3 (p‐Tyr219)	0.6	E2F1 (p‐Thr433)	0.6
IKK‐α/β (p‐Ser180/181)	1.7	c‐Jun (p‐Thr93)	0.6	PTEN (p‐Ser380)	0.6
IKK‐β (p‐Tyr199)	1.6	IKK‐ α/β (p‐Ser180/181)	0.6	Pyk2 (p‐Tyr580)	0.6
Smad2 (p‐Ser250)	1.6	EEF2	0.7		
HDAC6 (p‐Ser22)	1.6	Elk1 (p‐Ser389)	0.7		
p27Kip1 (p‐Ser10)	1.6				
FAK (p‐Tyr576)	1.5				
SHP‐2 (p‐Tyr542)	1.5				
MAP3K7/TAK1 (p‐Thr184)	1.5				
JNK1/2/3 (p‐Thr183/Tyr185)	1.5				
HDAC5 (p‐Ser259)	1.5				
Smad1 (p‐Ser187)	1.5				
Tau (p‐Thr212)	0.5				
ACC1 (p‐Ser80)	0.6				

*Puromycin*					
Paxillin (p‐Tyr31)	2	MEK2 (p‐Thr394)	2.5	p44/42 MAPK (p‐Tyr204)	1.7
SMC1 (p‐Ser957)	1.9	Synuclein α (p‐Tyr125)	1.7	GATA1 (p‐Ser142)	1.6
GRK1 (p‐Ser21)	1.8	VAV1 (p‐Tyr174)	1.7	PAK1/2/3 (p‐Thr423/402/421)	0.5
HDAC6 (p‐Ser22)	1.5	P70S6K (p‐Ser424)	0.4	P73 (p‐Tyr99)	0.6
ETK (p‐Tyr40)	1.8	Elk1 (p‐Thr417)	0.5	Merlin (p‐Ser10)	0.6
MAP3K7/TAK1 (p‐Thr184)	1.5	EGFR (p‐Tyr869)	0.5	Estrogen Receptor‐α (p‐Ser104)	0.6
NFkB‐p65 (p‐Thr254)	1.8	p27Kip1 (p‐Thr187)	0.5	Tyrosine Hydroxylase (p‐Ser19)	0.6
P70S6K (p‐Ser371)	1.5	MKK4/SEK1 (p‐Ser80)	0.6	EGFR (p‐Tyr869)	0.6
IRS‐1 (p‐Ser794)	0.5	Catenin β (p‐Ser37)	0.6	ATF2 (p‐Ser62/44)	0.6
BAD (p‐Ser91/128)	0.7	Myc (p‐Thr358)	0.6	Tau (p‐Thr212)	0.6
		c‐Jun (p‐Thr93)	0.6	GATA1 (p‐Ser310)	0.6
		Ephrin B2 (p‐Tyr330)	0.7	IkB‐epsilon (p‐Ser22)	0.6
		CDK5 (p‐Tyr15)	0.7	Pyk2 (p‐Tyr580)	0.6
		CD5 (p‐Tyr453)	0.7	DAPP1 (p‐Tyr139)	0.7
				p53 (p‐Ser20)	0.7
				P95/NBS1 (p‐Ser343)	0.7
				p38 MAPK (p‐Tyr182)	0.7

*NPAA*					
Paxillin (p‐Tyr31)	1.8	GluR1 (p‐Ser849)	1.9	SLP‐76 (p‐Tyr128)	1.9
GSK3 α (p‐Ser9)	1.7	VASP (p‐Ser157)	1.7	Caspase 9 (p‐Ser196)	1.6
HER3/ErbB3 (p‐Tyr1222)	1.6	HSP27 (p‐Ser82)	1.5	Cortactin (p‐Tyr421)	0.6
Progest. Receptor (p‐Ser190)	1.6	ATF2 (p‐Ser62/44)	0.4	P73 (p‐Tyr99)	0.6
FAK (p‐Tyr576)	1.5	EGFR (p‐Tyr869)	0.5	AKT1 (p‐Tyr474)	0.6
c‐Jun (p‐Ser63)	1.5	ATF1 (p‐Ser63)	0.6	DAPP1 (p‐Tyr139)	0.6
CDK7 (p‐Thr170)	0.6	BCR (p‐Tyr177)	0.6	Raf1 (p‐Ser296)	0.6
IRS‐1 (p‐Ser794)	0.6	MKK4/SEK1 (p‐Ser80)	0.6	BCR (p‐Tyr177)	0.6
Chk1 (p‐Ser286)	0.6	Ephrin B2 (p‐Tyr330)	0.6	PTEN (p‐Ser380)	0.6
		CDK5 (p‐Tyr15)	0.6	Tyrosine Hydroxylase (p‐Ser19)	0.7
		CD5 (p‐Tyr453)	0.6		
		MKK7/MAP2K7 (p‐Thr275)	0.6		
		IKK‐α/β(p‐Ser180/181)	0.6		
		Catenin β (p‐Tyr489)	0.7		
		Myc (p‐Thr358)	0.7		

FC, fold change.

### 3.2 IHC analysis of MEK2 (pThr394) expression in kidney tissue

**Figure 1:**
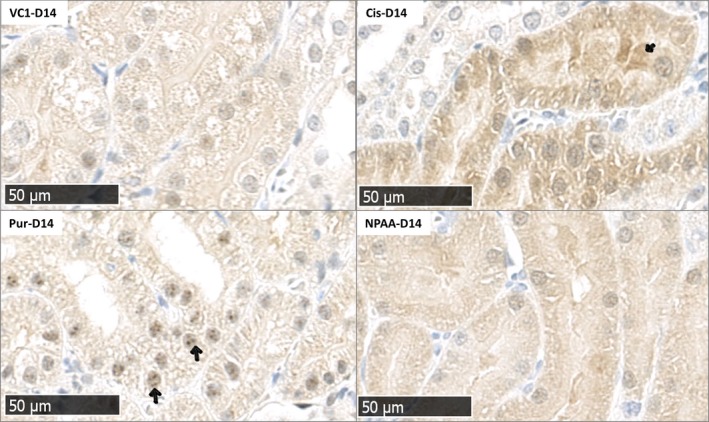
Immunohistochemical analysis of MEK2 (pThr394) in rat kidney tissue on day 14 after cisplatin (Cis‐D14), puromycin (Pur‐D14), NPAA (NPAA‐D14) or vehicle control administration (VC1‐D14), bar = 50 µm. Arrows indicate increased nuclear staining in the dilated tubules after puromycin treatment (Pur‐D14) and increased cytoplasmic staining in the tubule after cisplatin treatment (Cis‐D14)

We selected MEK2 (pThr394) to further investigate its expression in kidney tissue by IHC. No changes were observed in MEK2 (pThr394) phosphorylation for any treatment compared to control on day 7 after drug administration (data not shown). On day 14, an increase in MEK2 (pThr394) phosphorylation was detected after cisplatin and puromycin administration. Increased nuclear staining was observed in dilated tubules after puromycin treatment and cytoplasmic staining in the affected tubules after cisplatin treatment (Figure 1).

### 3.3 Analysis of differentially expressed phosphoproteins

Based on the phosphorylation sites of the selected phosphoproteins, we determined their implication in different biological processes by PhosphoSitePlus (Figure S1). Main processes affected in all treatments on day 7 were apoptosis and cytoskeletal reorganization. In addition, cisplatin induced inflammation on day 7, apoptosis on day 14 and cell growth and proliferation on day 28. In puromycin‐induced injury, inhibition of proliferation on day 14, and induction of apoptosis and cytoskeletal reorganization on day 28 could be observed. NPAA‐induced injury was associated with inhibition of transcription on days 7 and 14, and cell growth on day 28.

## 4 DISCUSSION

Available data on phosphoproteins dysregulation in the context of kidney injury are scarce and focuses mainly on MAPK activation in AKI.^2^ Here, we analysed three rat models of DIKI using phosphoprotein microarrays. Cisplatin, puromycin and NPAA were selected based on their ability to induce proximal tubule, glomerular and collecting duct injury respectively.^5^, ^6^, ^7^ This allowed us to identify phosphoprotein signatures associated with region‐specific kidney injury.

Cisplatin‐induced injury on day 7 resulted in altered phosphorylation of p53, MAPK3K7, JNK1, NFkB and IKK. Previously, activation of p53 was associated with tubular apoptosis in cisplatin‐induced injury in mice.^8^ It was also shown that cisplatin‐induced activation of ERK p38 and JNK/SAPK in vivo preceded the development of AKI.^9^ Phosphorylation of p38‐MAPK and NF‐κB was found to be up‐regulated in rat kidneys following gentamicin treatment.^10^ In our cisplatin‐induced model, the number of dysregulated phosphoproteins decreased over time (day 28) and was mostly represented by proteins involved in cell growth and proliferation. This might be a consequence of a recovery from injury after a single administration of cisplatin confirmed by the lack of histopathological lesions in proximal tubules on day 28.^5^

Contrary to cisplatin, daily administration of puromycin resulted in the number of dysregulated phosphoproteins increasing over time. On day 28, the phosphoproteins involved in processes like apoptosis, cytoskeletal reorganization and proliferation were up‐regulated. According to histopathology findings, daily administration of puromycin led to severe injury to the glomerulus and tubular damage, which is secondary to glomerular injury (day 14 and 28). This might explain some overlap between up‐ and down‐regulated phosphoproteins in cisplatin and puromycin (day 14). For MEK2 (pThr394), we confirmed an increased expression in affected tubules after cisplatin and puromycin injury on day 14 by IHC.

Daily administration of NPAA also induced significant changes in phosphoprotein expression which were related to apoptosis and cytoskeletal reorganization. Interestingly, on day 14, several phosphoproteins involved in transcription were down‐regulated. We also observed up‐regulation of phosphorylation of metabotropic glutamate receptor 1 (mGluR1), which is part of glutamatergic system in the brain. Interestingly, renal receptors for glutamate, including mGluR1, were shown to be modulated in AKI.^11^

In summary, we established DIKI‐associated phosphoprotein signatures in the rat by using kidney tissue and antibody‐based arrays. These phosphoprotein profiles varied depending on a time‐point and a nephron‐specific injury analyzed. Altered phosphoprotein levels were confirmed for pMEK (Thyr394) by IHC. Linking phosphoprotein changes and biological processes in the kidney upon injury helps to understand different molecular mechanisms underlying kidney damage/recovery. Selected candidates can be used for further investigation and validation as mechanistic biomarkers in vitro models.

## CONFLICT OF INTEREST

The authors confirm that there are no conflicts of interest.
