# Supplementary figures and images for: Phosphoprotein expression profiles in rat kidney injury: Source for potential mechanistic biomarkers
Source: J Cell Mol Med. 2019 Jan 12;23(3):2251–5. doi: 10.1111/jcmm.14103 (PMC6378196; doi:10.1111/jcmm.14103)

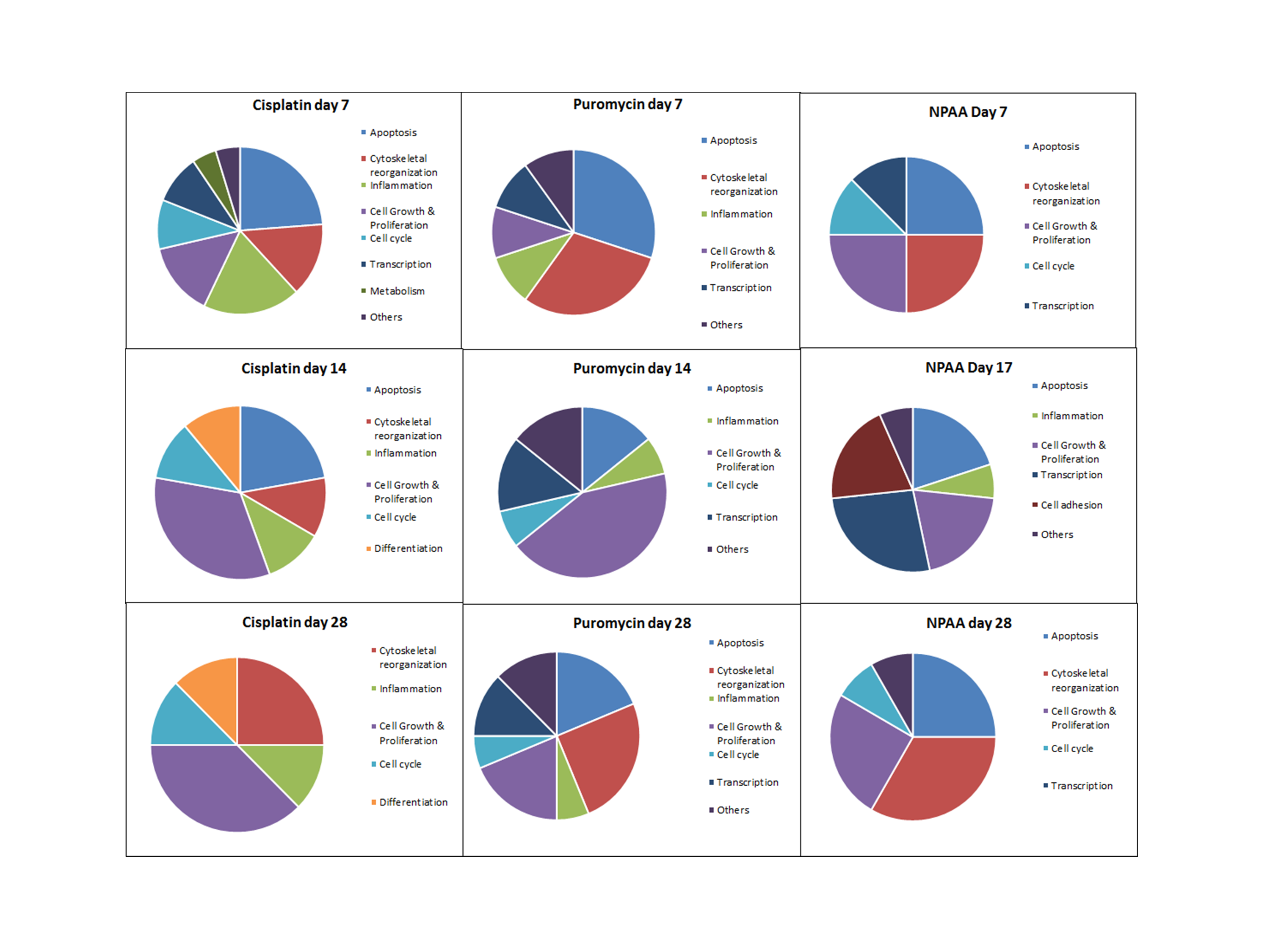

Supplement: Supplementary file 1 [file JCMM-23-2251-s001.tif]
