# Supplementary material for: Phosphoprotein expression profiles in rat kidney injury: Source for potential mechanistic biomarkers
Source: J Cell Mol Med. 2019 Jan 12;23(3):2251–5. doi: 10.1111/jcmm.14103 (PMC6378196; doi:10.1111/jcmm.14103)
Supplement: Supplementary file 2 [file JCMM-23-2251-s002.docx]

**Supplementary data**

**Phosphoprotein expression profiles in rat kidney injury: source of potential mechanistic biomarkers**

***Gryshkova Vitalina^1^*********, Cotter Mabel^3^, Portia McGhan^4^, Jana Obajdin^5^, Renaud Fleurance^1^, Nogueira da Costa Andre^2^***

*^1^Investigative Toxicology, Development Science, UCB Biopharma SPRL, Chemin du Foriest 1, B-1420 Braine L'Alleud, Belgium*

*^2^Experimental Medicine and Diagnostics, Translational Medicine, UCB Biopharma SPRL, Chemin du Foriest 1, B-1420 Braine L'Alleud, Belgium*

*^3^Safety & Environmental Assurance Centre, Unilever U.K., Unilever House, Blackfriars, London, EC4Y 0DY, UK*

*^4^MRC Institute of Genetics & Molecular Medicine, The University of Edinburgh, Western General Hospital, Crewe Road, Edinburgh EH4 2XU*

*^5^Centre for Stem Cells & Regenerative Medicine King's College London 28th Floor, Tower Wing Guy's Campus Great Maze Pond London SE1 9RT*

*** *To whom correspondence should be addressed at Investigative Toxicology, Development Science, UCB Biopharma SPRL, Chemin du Foriest 1, Building R9, B-1420 Braine L'Alleud, Belgium,* [*tel:+3223862619*](tel:+3223862619)*,* [Vitalina.gryshkova@ucb.com](mailto:Vitalina.gryshkova@ucb.com)

**Table S1**. Phosphoproteins dysregulated in kidney injury induced by cisplatin, puromycin or NPAA on day 7 after drug administration.

| **Phosphoprotein** | **FC**  **Cisplatin** | **p-value** | **FC**  **Puromycin** | **p-value** | **FC**  **NPAA** | **p-value** |
| --- | --- | --- | --- | --- | --- | --- |
| p53 (p-Ser378) | ***2,0*** | 0,003 | ***1,9*** | 0,14 | 1,2 | 0,39 |
| Rac1/cdc42 (p-Ser71) | ***1,9*** | 0,06 | ***2,2*** | 0,29 | 1,0 | 0,92 |
| NFkB-p65 (p-Ser468) | ***1,8*** | 0,05 | ***1,7*** | 0,17 | ***1,5*** | 0,19 |
| GSK3 beta (p-Ser9) | ***1,8*** | 0,04 | ***1,8*** | 0,13 | ***1,7*** | 0,03 |
| IRS-1 (p-Ser323) | ***1,8*** | 0,05 | ***1,6*** | 0,23 | ***1,5*** | 0,23 |
| IKK-alpha/beta (p-Ser180/181) | ***1,7*** | 0,05 | ***1,5*** | 0,31 | 1,2 | 0,44 |
| SMC1 (p-Ser957) | ***1,7*** | 0,01 | ***1,9*** | 0,05 | ***1,6*** | 0,18 |
| GRK1 (p-Ser21) | ***1,7*** | 0,24 | ***1,8*** | 0,05 | 1,4 | 0,03 |
| LCK (p-Tyr504) | ***1,7*** | 0,05 | 1,3 | 0,23 | 1,1 | 0,63 |
| Smad2 (p-Ser250) | ***1,6*** | 0,03 | 1,4 | 0,07 | 1,4 | 0,16 |
| HDAC6 (p-Ser22) | ***1,6*** | 0,04 | ***1,5*** | 0,02 | 1,3 | 0,12 |
| ETK (p-Tyr40) | ***1,6*** | 0,24 | ***1,8*** | 0,05 | 1,4 | 0,29 |
| IKK-beta (p-Tyr199) | ***1,6*** | 0,04 | 1,3 | 0,34 | 1,0 | 0,87 |
| p27Kip1 (p-Ser10) | ***1,6*** | 0,05 | ***1,6*** | 0,15 | 1,3 | 0,06 |
| SHP-2 (p-Tyr542) | ***1,5*** | 0,05 | 1,4 | 0,16 | 1,2 | 0,48 |
| MAP3K7/TAK1 (p-Thr184) | ***1,5*** | 0,05 | ***1,5*** | 0,05 | 1,2 | 0,24 |
| JNK1/2/3 (p-Thr183/Tyr185) | ***1,5*** | 0,03 | ***1,5*** | 0,20 | 1,3 | 0,12 |
| NFkB-p65 (p-Thr254) | ***1,5*** | 0,16 | ***1,8*** | 0,04 | 1,2 | 0,36 |
| HDAC5 (p-Ser259) | ***1,5*** | 0,05 | ***1,7*** | 0,33 | 1,3 | 0,29 |
| FAK (p-Tyr576) | ***1,5*** | 0,05 | ***1,7*** | 0,15 | ***1,5*** | 0,004 |
| Smad1 (p-Ser187) | ***1,5*** | 0,05 | ***1,6*** | 0,19 | 1,3 | 0,23 |
| c-Jun (p-Ser63) | 1,4 | 0,11 | ***1,6*** | 0,25 | ***1,5*** | 0,05 |
| HER3/ErbB3 (p-Tyr1222) | 1,4 | 0,41 | ***1,6*** | 0,15 | ***1,6*** | 0,05 |
| Paxillin (p-Tyr31) | 1,3 | 0,27 | ***2,0*** | 0,05 | ***1,8*** | 0,04 |
| P70S6K (p-Ser371) | 1,0 | 0,97 | ***1,5*** | 0,05 | 1,2 | 0,34 |
| Progesterone Receptor (p-Ser190) | 1,0 | 0,89 | ***1,7*** | 0,32 | ***1,6*** | 0,05 |
| BAD (p-Ser91/128) | 0,9 | 0,30 | ***0,7*** | 0,05 | 0,8 | 0,25 |
| IRS-1 (p-Ser794) | ***0,7*** | 0,24 | ***0,5*** | 0,05 | ***0,6*** | 0,05 |
| Chk1 (p-Ser286) | ***0,7*** | 0,07 | 0,9 | 0,52 | ***0,6*** | 0,04 |
| ACC1 (p-Ser80) | ***0,6*** | 0,04 | 0,8 | 0,11 | 0,9 | 0,70 |
| Tau (p-Thr212) | ***0,5*** | 0,05 | 0,9 | 0,77 | ***0,7*** | 0,16 |
| CDK7 (p-Thr170) | ***0,8*** | 0,22 | 0,7 | 0,15 | ***0,6*** | 0,05 |

*The cells with p-values ≤ 0.05 and fold changes FC ≥ 1.5 and FC ≤ 0.7 are marked in grey*

**Table S2.** Phosphoproteins dysregulated in kidney injury induced by cisplatin, puromycin or NPAA on day 14 after drug administration.

| **Phosphoprotein** | **FC**  **Cisplatin** | **p-value** | **FC**  **Puromycin** | **p-value** | **FC**  **NPAA** | **p-value** |
| --- | --- | --- | --- | --- | --- | --- |
| MEK2 (p-Thr394) | ***2,2*** | 0,01 | ***2,5*** | 0,01 | ***1,5*** | 0,13 |
| Synuclein alpha (p-Tyr125) | ***1,8*** | 0,01 | ***1,7*** | 0,002 | ***1,5*** | 0,13 |
| AKT1 (p-Thr308) | ***1,6*** | 0,04 | 1,3 | 0,11 | 0,9 | 0,10 |
| Src (p-Tyr529) | ***1,6*** | 0,05 | 1,1 | 0,48 | ***1,5*** | 0,39 |
| HSP27 (p-Ser82) | 1,1 | 0,48 | 1,0 | 0,81 | ***1,5*** | 0,05 |
| VASP (p-Ser157) | 1,1 | 0,78 | 1,0 | 0,98 | ***1,7*** | 0,04 |
| VAV1 (p-Tyr174) | 1,0 | 0,87 | ***1,7*** | 0,01 | ***1,6*** | 0,19 |
| ATF1 (p-Ser63) | 1,0 | 0,93 | 0,8 | 0,23 | ***0,6*** | 0,05 |
| EGFR (p-Thr693) | 0,9 | 0,75 | 0,9 | 0,63 | ***0,6*** | 0,05 |
| GluR1 (p-Ser849) | 0,9 | 0,84 | 1,1 | 0,76 | ***1,9*** | 0,03 |
| BCR (p-Tyr177) | 0,9 | 0,71 | 1,1 | 0,83 | ***0,6*** | 0,05 |
| Catenin beta (p-Tyr489) | 0,9 | 0,55 | 0,8 | 0,13 | ***0,7*** | 0,05 |
| MKK4/SEK1 (p-Ser80) | 0,8 | 0,53 | ***0,6*** | 0,002 | ***0,6*** | 0,003 |
| Catenin beta (p-Ser37) | 0,8 | 0,45 | ***0,6*** | 0,03 | 0,8 | 0,18 |
| Ephrin B2 (p-Tyr330) | 0,8 | 0,26 | ***0,7*** | 0,04 | ***0,6*** | 0,01 |
| CDK5 (p-Tyr15) | 0,8 | 0,33 | ***0,7*** | 0,05 | ***0,6*** | 0,03 |
| Elk1 (p-Thr417) | 0,8 | 0,05 | ***0,5*** | 0,01 | 1,3 | 0,18 |
| CD5 (p-Tyr453) | ***0,7*** | 0,13 | ***0,7*** | 0,01 | ***0,6*** | 0,01 |
| Myc (p-Thr358) | ***0,7*** | 0,16 | ***0,6*** | 0,05 | ***0,7*** | 0,06 |
| ATF2 (p-Ser62/44) | ***0,7*** | 0,26 | ***0,6*** | 0,25 | ***0,4*** | 0,05 |
| EEF2 (p-Thr56) | ***0,7*** | 0,04 | 0,8 | 0,19 | 1,0 | 0,76 |
| Elk1 (p-Ser389) | ***0,7*** | 0,02 | 0,8 | 0,25 | 0,9 | 0,17 |
| P70S6K (p-Ser424) | ***0,7*** | 0,25 | ***0,4*** | 0,05 | ***0,6*** | 0,12 |
| Claudin 3 (p-Tyr219) | ***0,6*** | 0,04 | 1,0 | 0,98 | ***0,7*** | 0,09 |
| MKK7/MAP2K7 (p-Thr275) | ***0,6*** | 0,10 | 0,8 | 0,27 | ***0,6*** | 0,05 |
| c-Jun (p-Thr93) | ***0,6*** | 0,001 | ***0,6*** | 0,06 | 0,9 | 0,44 |
| IKK-alpha/beta (p-Ser180/181) | ***0,6*** | 0,03 | ***0,7*** | 0,10 | ***0,6*** | 0,03 |
| EGFR (p-Tyr869) | ***0,5*** | 0,01 | ***0,5*** | 0,02 | ***0,5*** | 0,03 |
| p27Kip1 (p-Thr187) | ***0,5*** | 0,10 | ***0,5*** | 0,03 | 0,8 | 0,37 |

*The cells with p-values ≤ 0.05 and fold changes FC ≥ 1.5 and FC ≤ 0.7 are marked in grey*

**Table S3.** Phosphoproteins dysregulated in kidney injury induced by cisplatin, puromycin or NPAA on day 28 after drug administration.

| **Phosphoprotein** | **FC**  **Cisplatin** | **p-value** | **FC**  **Puromycin** | **p-value** | **FC**  **NPAA** | **p-value** |
| --- | --- | --- | --- | --- | --- | --- |
| c-Jun (p-Thr93) | ***1,7*** | 0,05 | 1,3 | 0,45 | ***2,1*** | 0,29 |
| p44/42 MAPK (p-Tyr204) | 1,3 | 0,15 | ***1,7*** | 0,05 | 1,4 | 0,10 |
| GATA1 (p-Ser142) | 1,2 | 0,37 | ***1,6*** | 0,05 | 1,1 | 0,59 |
| SLP-76 (p-Tyr128) | 1,1 | 0,79 | ***1,7*** | 0,13 | ***1,9*** | 0,05 |
| Caspase 9 (p-Ser196) | 1,0 | 0,91 | 1,3 | 0,17 | ***1,6*** | 0,05 |
| P73 (p-Tyr99) | 1,0 | 0,96 | ***0,6*** | 0,02 | ***0,6*** | 0,03 |
| Merlin (p-Ser10) | 1,0 | 0,97 | ***0,6*** | 0,05 | 1,3 | 0,32 |
| AKT1 (p-Tyr474) | 0,9 | 0,71 | 0,8 | 0,29 | ***0,6*** | 0,05 |
| Cortactin (p-Tyr421) | 0,9 | 0,78 | 0,8 | 0,60 | ***0,6*** | 0,03 |
| Estrogen Receptor-alpha (p-Ser104) | 0,9 | 0,37 | ***0,6*** | 0,01 | 1,1 | 0,66 |
| Tyrosine Hydroxylase (p-Ser19) | 0,9 | 0,74 | ***0,6*** | 0,03 | ***0,7*** | 0,05 |
| EGFR (p-Tyr869) | 0,9 | 0,77 | ***0,6*** | 0,02 | 1,1 | 0,72 |
| DAPP1 (p-Tyr139) | 0,9 | 0,34 | ***0,7*** | 0,05 | ***0,6*** | 0,01 |
| p53 (p-Ser20) | 0,8 | 0,35 | ***0,7*** | 0,05 | 0,9 | 0,50 |
| Raf1 (p-Ser296) | 0,8 | 0,36 | 0,8 | 0,08 | ***0,6*** | 0,05 |
| ATF2 (p-Ser62/44) | ***0,7*** | 0,14 | ***0,6*** | 0,01 | 0,8 | 0,33 |
| P95/NBS1 (p-Ser343) | ***0,7*** | 0,07 | ***0,7*** | 0,02 | 0,8 | 0,05 |
| BCR (p-Tyr177) | ***0,7*** | 0,10 | 0,8 | 0,24 | ***0,6*** | 0,05 |
| Tau (p-Thr212) | ***0,7*** | 0,26 | ***0,6*** | 0,05 | ***0,6*** | 0,08 |
| p38 MAPK (p-Tyr182) | ***0,7*** | 0,10 | ***0,7*** | 0,04 | 0,9 | 0,66 |
| GATA1 (p-Ser310) | ***0,6*** | 0,05 | ***0,6*** | 0,03 | 0,8 | 0,32 |
| E2F1 (p-Thr433) | ***0,6*** | 0,05 | ***0,7*** | 0,11 | 0,9 | 0,36 |
| PTEN (p-Ser380) | ***0,6*** | 0,05 | ***0,6*** | 0,08 | ***0,6*** | 0,05 |
| IkB-epsilon (p-Ser22) | ***0,6*** | 0,15 | ***0,6*** | 0,03 | ***0,7*** | 0,07 |
| Pyk2 (p-Tyr580) | ***0,6*** | 0,04 | ***0,6*** | 0,002 | 0,8 | 0,34 |
| IKK-alpha (p-Thr23) | ***0,5*** | 0,05 | ***0,5*** | 0,10 | ***0,6*** | 0,11 |
| PAK1/2/3 (p-Thr423/402/421) | ***0,5*** | 0,05 | ***0,5*** | 0,05 | ***0,6*** | 0,07 |
| P70S6K (p-Ser371) | ***0,5*** | 0,002 | ***0,7*** | 0,24 | ***0,6*** | 0,07 |

*The cells with p-values ≤ 0.05 and fold changes FC ≥ 1.5 and FC ≤ 0.7 are marked in grey*


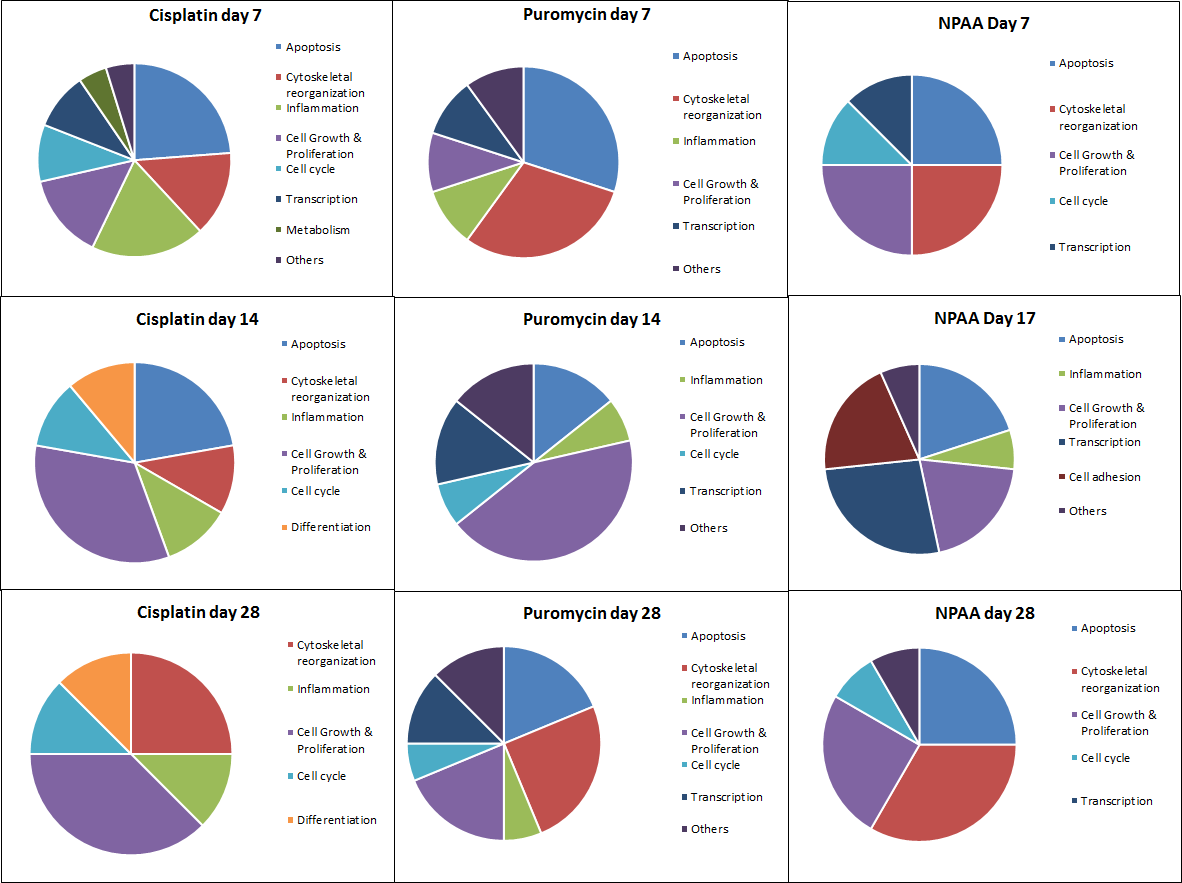


**Figure S1.** Pie charts of the main biological processes disrupted in kidney injury based on phosphoprotein profiling.
